# Is cannabidiol (CBD) effective to ease separation anxiety?

**DOI:** 10.1016/j.heliyon.2024.e25851

**Published:** 2024-02-05

**Authors:** Nobuo Masataka

**Affiliations:** Center for Research of Developmental Disorders, Kyoto, Japan

**Keywords:** Anxiety, Cannabidiol, CBD, Fear, Attachment, Domestic cat, Separation

## Abstract

CBD is the primary noneuphorizing and nonaddictive compound of cannabis. It has recently been shown to possess considerable therapeutic potential for treating a wide range of neuropsychiatric disorders in humans, such as anxiety. In addition to humans, domestic cats are provided with such endocannabinoid system with which CBD interacts almost in the same manner researchers think it does in humans. However, little has been known about the clinical implications of CBD in the animals. Here the effects of CBD administration upon separation anxiety were evaluated in ten healthy cats. The animals experienced brief separation from their caregivers twice, once following the administration of CBD 4.0 mg/kg/day over a 2-week-period and once following the administration of sunflower oil alone as placebo. Upon the caregiver's return from a brief absence, response of the animal can be categorized into these three categories: reduced stress one with contact-exploration balance with the caregiver (the Secure Base Effect), remaining stressful and keeping proximity excessively, and avoidance behavior or approach/avoidance conflict (disorganized behavior). When receiving placebo administration, the cats spent more time in physical contact with the caregiver or, in avoidant behavior than when receiving CBD administration whereas they spent more time in proximity to the caregiver when receiving CBD administration than when receiving placebo (ps < 0.01). With the brief separation from their caregivers, the cats became distressed, and signs of such distress was more evident after the placebo administration whereas the Secure Base Effect was more distinct in the results of SBT when receiving CBD administration than when receiving placebo. The results suggest anxiety-reducing effects of CBD in cats.

## 1 Introduction

In the mid-20th century, a US psychologist conducted a pioneering study that investigated the response of infant macaques when separated from their mothers and placed alone [1]. When separated, they were found to be extremely distressed. However, if a surrogate mother made from cloth was presented, such distress was eased, and the infants used the surrogate mother as “a source of security”. Since the study, such effects have been referred to as “the security base effect (SBE)” upon the infants’ separation anxiety.

Up to the present, SBE has been systematically evaluated with a number of nonhuman animals, including domestic cats, on the basis of the results of the secure base test (SBT). The current study was conducted to investigate whether cannabidiol (CBD) was effective to ease the separation anxiety by evaluating the results of SBT.

CBD is the primary noneuphorizing and nonaddictive compound of cannabis. In humans, recently, it has been shown to possess considerable therapeutic potential for treating a wide range of neuropsychiatric disorders. They include chronic pain [2], nausea [3], epilepsy [4]. Psychosis [5], and anxiety [6,7]. The interaction of CBD with the serotonin receptor exerted analgesic and anxiolytic effects [8].

In addition to humans, both dogs and cats are provided with such endocannabinoid system with which CBD interacts almost in the same manner researchers think it does in humans [9,10]. Consequently, the use of CBD-rich hemp products is becoming popular among pet owners.

Domestic cats as well as dogs, raised with humans, are known to form attachments with their caregivers and other humans with whom they form a stable relationship [[11], [12], [13]]. As already noted. how securely the animals are attached to such human has been measured so far by using SBT.

During typical testing of SBT, each animal is to spend for a given length of time in a novel room with their caregiver, first. That is followed by a 2-min alone phase, and then a 2-min reunion phase continues. After the caregiver's return, response of the animal can be classified into these three categories as summarized in the previous literature [12]: “reduced stress one with contact-exploration balance with the caregiver (SBE), remaining stressful and keeping proximity excessively, and avoidance behavior or approach/avoidance conflict (disorganized behavior)”. The author hypothesized that if CBD administration exerts anxiety-reducing effects in cats under study, SBE would be more distinct in the results of SBT, when they received CBD administration as compared with when they received administration of its placebo.

## 2 Material and methods

### *2* Participants

Ten healthy domestic cats and their caregivers were recruited through personal contact, and advertisement on a local newspaper that was published in Nago, Okinawa Prefecture, Japan where this research was conducted. The cats were all males and had been neutered prior to study, ranging in age from 3 to 5 years. They were all mixed breeds. A veterinarian performed physical examinations on all participants at the start of the study and weekly thereafter. The physical examinations revealed no abnormalities or weight alternations, and neither inappetence nor behavioral changes were reported in any of the subjects during this investigation.

### 2.2 Design and administration

During the study, the participants received both a 2-week-administration of CBD and a 2-week-administration of matching placebo. Both were conducted, being separated from one another with a 30-day-interval.

Domestic cats as well as dogs, raised with humans, are known to form attachments with their caregivers and other humans with whom they form a stable relationship. How securely the animals are attached to such human has been measured so far by using SBT. In order to evaluate the effects of the administration upon the results of SBT, each animal was tested on the day when each administration was completed.

At the commencement of the present research, 5 of the 10 cats were assigned to receive the CBD administration prior to the placebo administration while the placebo was to be administered to the remaining 5 cats prior to the CBD administration. For the CBD administration, CBD at 4.0 mg/kg was daily administered with sunflower oil as carrier in the morning over a 2-week period. For the placebo administration, the cats were to daily receive sunflower oil alone as a matching placebo over the other 2-week period.

On a day when the CBD or the placebo was administered, a veterinary technician employed for this study visited the cats at the house of their caregivers with CBD oil or placebo before daily feeding, confirmed that the cat was healthy, and administered the necessary amount of the prepared oil to the participant, using a syringe. The technician did not know about the purpose of the present research.

The CBD used was Elixinol-Entry Hemp Oil (the product of Elixinol, US) that was produced from the stalk of hemp plants. A 10-ml-bottle of the product that was for sale by the company contained 500 mg of CBD (50.0 mg/ml), but no delta-9-tetrahydrocannabinol (THC). The placebo contained sunflower oil. For each cat, roughly 40 ml of the CBD oil with sunflower oil or the same amount of the placebo was re-bottled in a container that was different from that in which it had been originally bottled, and that was identical in size, color, smell, and as well as taste. The technician who visited the home of the guardian did not know the content of the container.

### 2.3 SBT session

With respect to each cat, SBT session was undertaken in the testing area of a house rented for this study where its caregiver visited with the cat. With regerd to the detailed setup of the testing, the author followed the previous method of published feline and canid attachment study [12,13]. The testing area was a room measuring 3.5 m by 4.5 m. The room, sparsely furnished, was provided with two doors without any window, one of which was used for entrance and exit. Along a wall roughly 2 m distant from the door, there located a chair, around which a circle with a 1 m radius was marked with tape. From the ceiling of the room two were hung two video cameras that covered its entire floor. Three toys were prepared on the floor. This room as well as layout was novel to the cat as well as to caregiver prior to the current experiment.

When arriving at the house, the caregiver met the experimenter with its cat at the entrance hall. Subsequently, the cat and caregiver was led into the testing area. As noted in the previous study [12]. The caregiver was asked to sit back down in the chair, and instructed that “when the cat entered the circle surrounding the chair, they could play with your cat there as usual, but the cat was outside the circle, they must remain silent, passive, and non-moving (no talking, no gestures). The video in the room was kept on.” As previously reported [12], “the SBT was divided into three conditions, each lasting 2 min. *Baseline condition:* after giving instructions, the experimenter exited the room and closed the door, leaving the cat and caregiver in the testing area for a following 2-min period. *Alone condition:* when 2 min elapsed, the experimenter opened the door, and this time indicated to the caregiver to stand up, say “goodbye” to the cat, exit the room.” The caregiver was ordered to follow the experimenter, who left the room. Consequently, the cat was left alone. For the cat, that would serve as a mild stressor, “which would allow for assessment of the secure base effect during the return condition” [12]. *Return condition:* 2 min later, the caregiver was ordered to come back to the room and take the seat whom the experimenter did not follow. The instructions for the caregiver were the same as for those in the baseline condition.

Cats’ behavior that was videotaped during SBT was coded into the following three categories, as in the previous study [12], with respect to the caregiver: “(1) in physical contact with the caregiver, (2) in proximity to the caregiver (within the circle with a 1-m radius around the caregiver) without any physical contact, and (3) in avoidant behavior (outside the circle). When the animal stayed in proximity to the caregiver without physical contact, the author determined that the SBE was observed”.

## 3 Results

Table 1 presents the total duration of the SBE recorded as well as of the other two behavior categories exhibited by the 10 cats during return condition, when they received the administration with CBD and when they received the administration with placebo. When receiving placebo administration, the cats spent more time in physical contact with the caregiver or, in avoidant behavior than when receiving CBD administration (ps < 0.01, binomial tests) whereas they spent more time in proximity to the caregiver when receiving CBD administration than when receiving placebo (p < 0.01). SBE was more distinct when receiving CBD administration than when receiving placebo administration.

**Table 1:** Total duration (in sec) of videorecorded behaviors of the ten cats during return condition, when receiving CBD administration and placebo administration, that were coded into the following three categories in relation to the caregiver: (1) in physical contact with the caregiver, (2) in proximity to the caregiver (within the circle with a 1-m radius around the caregiver) without any physical contact, and (3) in avoidant behavior (outside the circle).

Cat	Administration					
	CBD			Placebo		
	Contact	Proximity	Avoidant	Contact	Proximity	Avoidant
1	20.6	67.4	12.0	22.6	63.2	14.2
2	16.1	72.8	11.1	17.2	68.8	11.7
3	23.4	59.6	16.0	23.2	63.0	13.8
4	18.8	68.4	12.8	21.6	64.3	14.1
5	27.9	55.3	16.8	30.4	51.1	18.5
6	9.6	71.0	19.4	11.7	68.6	19.7
7	11.3	82.1	6.9	10.0	77.3	12.7
8	14.5	60.3	25.2	19.6	65.2	15.2
9	12.0	65.7	12.3	18.5	60.9	29.6
10	11.7	66.5	21.9	14.4	62.1	23.4
Mean	16.6	66.9	15.4	18.9	64.5	16.4

When videotaped cats’ behavior during baseline condition was coded according to the similar protocol, on the other hand, the total duration (in sec) when the cats spent in proximity to the caregiver did not differ statistically significantly whether they received CBD administration (mean = 51.2 s) or they received placebo administration (mean = 54.9 s) (p > 0.20).

## 4 Discussion

A recent study [13] reports the presence of a similar capacity in cats for the formation of secure and insecure attachments towards human caregivers as has been demonstrated in human children. Results of the present study also confirm such finding. They suggest that, in general, the participant cats spent a relatively great proportion of time in proximity to their caregivers rather than in physical contact or exhibiting avoidant behavior, showing SBE. However, the degree of such effects could be somehow modulated, and be more distinct when CBD was administered to the cats than when placebo was administered. After the brief separation from their caregivers, the participants became distressed, and signs of such distress became more evident after the placebo administration. With CBD administration, they became more secured upon such separation from their caregivers. The results appear to be similar to anxiety-reducing effects of CBD that have been reported in humans.

A pioneering study [14] investigated effects of CBD on people with social anxiety disorder (SAD) who were public speaking, and found that those people who were treated with CBD had reduced anxiety and improved comfort when they were speaking. More recently, the author recruited 37 Japanese teenagers with SAD who were randomly allocated to receive treatment with either CBD or a placebo for 4 weeks treatment [15]. The placebo treatment contained olive oil while the CBD treatment contained 300 mg of CBD oil. When anxiety was measured before and after treatment, the teenagers who received the treatment with CBD had significantly lower scores in both of the anxiety scales when compared with the teenagers who received the placebo treatment. The present results are quite consistent with the findings reported in these two studies.

As one of the possible explanations for the present results, taken together, the author hypothesizes anxiety-reducing effects of CBD in healthy domestic cats, as has been reported in humans [8,16,17]. Besides humans, CBD has been shown to reduce anxious behaviours exclusively in rodents [18,19].

More recently, such effects have been reported in domestic dogs, [20]. In the study, ten 4- to 7-year-old healthy dogs experienced temporary separation from their caregivers twice, once following the administration of CBD at 2.0 mg/kg/day over a 2-week-period, and once following the administration of the same amount of olive oil as placebo over the same length of period. When the behavioral assessment was conducted by computing the total duration of any vocal activity exhibited by the dogs before the separation from their caregivers and after the separation, it was found that all of the 10 participant dogs vocalized more often when being left alone (after the separation) than when being with their caregivers (before the separation), whether they had received the administration of CBD or placebo. Following the CBD administration, however, the degree of such increase was significantly less robust than that following the placebo administration.

To my best knowledge, the current study is the first demonstration of the anxiolytic effects of CBD on feline response to a brief separation. The implications of such findings cannot be dismissed when considering CBD as a useful option to treat anxiety that is a significant concern for a number of cat owners. Nevertheless it appears necessary to keep in mind that all separation-related problems are not caused by anxiety or fear because destruction behaviour can be motivated by overactivity, play, or boredom [21]. Such reasoning may lead us to assume that sedative effects of CBD could be exerted in healthy domestic dogs. This should apparently be the issue to be investigated in more detail in the near future.

## Statement of ethics

Study approval statement: This study protocol was reviewed and approved by the Institutinal Ethics Committee of Center for Study of Developmental Disorders, approval number [21-04].

Consent to participate statement: When conducting this study, written informed consent was obtained from all the caregivers of the dogs.

## Funding

This research was supported by the Grant of Excellent Graduate Schools program from MEXR, Japan (B-12). The funder had no role in study design, data collection and analysis, decision to publish, or preparation of the manuscript.

## Data availability statement

All datasets generated for this study will be available on request.

## Additional information

No additional information is available for this paper.

## CRediT authorship contribution statement

**Nobuo Masataka:** Writing – review & editing, Writing – original draft, Supervision, Project administration, Methodology, Investigation, Formal analysis, Data curation, Conceptualization.

## Declaration of competing interest

The authors declare that they have no known competing financial interests or personal relationships that could have appeared to influence the work reported in this paper.
